# Remote follow-up for pediatric epilepsy: effective care with equity-focused oversight

**DOI:** 10.3389/fpubh.2025.1727725

**Published:** 2025-12-05

**Authors:** Tiantian Zhong, Jiao Lei

**Affiliations:** 1 West China Second University Hospital, Sichuan University, Chengdu, China; 2 Key Laboratory of Birth Defects and Related Diseases of Women and Children (Sichuan University), Ministry of Education, Chengdu, China

**Keywords:** pediatric epilepsy, remote follow-up, telemedicine, digital public health, equity, key performance indicators, data protection, implementation science

## Abstract

**Background:**

Pediatric epilepsy care is often constrained by follow-up bottlenecks—travel burden, caregiver time loss, and limited pediatric neurology capacity—leading to fragmented management and preventable utilization. Methods: We conducted a narrative mini-review of studies and practice statements (2019–2025) identified in PubMed and Scopus, prioritizing pediatric populations and major guidelines relevant to tele-follow-up, adherence and refills, unplanned care, equity, privacy, data governance, and implementation; no meta-analysis was performed.

**Results:**

Evidence indicates that, for established patients, video or telephone visits can safely support medication titration, adverse-effect checks, seizure-diary review, counseling, and care coordination, with performance broadly comparable to clinic visits for these tasks when used judiciously. Tele-follow-up is inappropriate for new diagnoses, acute neurologic change, or other red flags requiring examination and electroencephalography. Effective implementation relies on risk-stratified schedules, predefined escalation thresholds and time-to-action, nurse-led triage, and measured substitution rather than wholesale replacement of in-person care. Equity risks persist for families with low connectivity, low income, language barriers, or disability; mitigations include device and data subsidies, school-linked access points, multilingual materials, and privacy-by-design with developmentally appropriate consent and auditable safeguards.

**Conclusion:**

Tele-follow-up is a feasible complement to clinic care that can reduce burden while maintaining quality when paired with transparent oversight. We propose a practical oversight approach using key performance indicators with equity stratifiers to monitor completion, adherence, and unplanned care, and outline research priorities on longer-term outcomes in under-connected populations, privacy impact evaluations, interoperability, and pragmatic trials with equity endpoints.

## 1 Introduction

Pediatric epilepsy creates lifelong burden for children, families, and health systems—especially in low- and middle-income countries where diagnostic and treatment gaps persist. Follow-up is often the bottleneck: long travel and time away from school or work, out-of-pocket transport costs, caregiver time loss, and limited pediatric neurology capacity. These frictions delay care after the first seizure and fragment management, driving preventable utilization and worse outcomes (1–4). Tele-follow-up and hybrid clinics connect in-person and remote care. For established patients, video or phone visits can support medication titration, adverse-effect checks, seizure-diary review, comorbidity screening, counseling, and care coordination; they should be avoided for new diagnoses, acute neurologic change, or other red flags that require examination and electroencephalography (EEG). Major professional bodies endorse telehealth in defined contexts, and pandemic experience accelerated its implementation in epilepsy services (1–3, 5, 6). Recent studies and practice statements suggest performance comparable to in-person care for several follow-up tasks when used judiciously, while underscoring limits around examination quality and EEG access (7, 8). This mini-review synthesizes effectiveness signals and equity risks and proposes KPI-based oversight to enable safe, scalable tele-follow-up.

**Figure 1:**
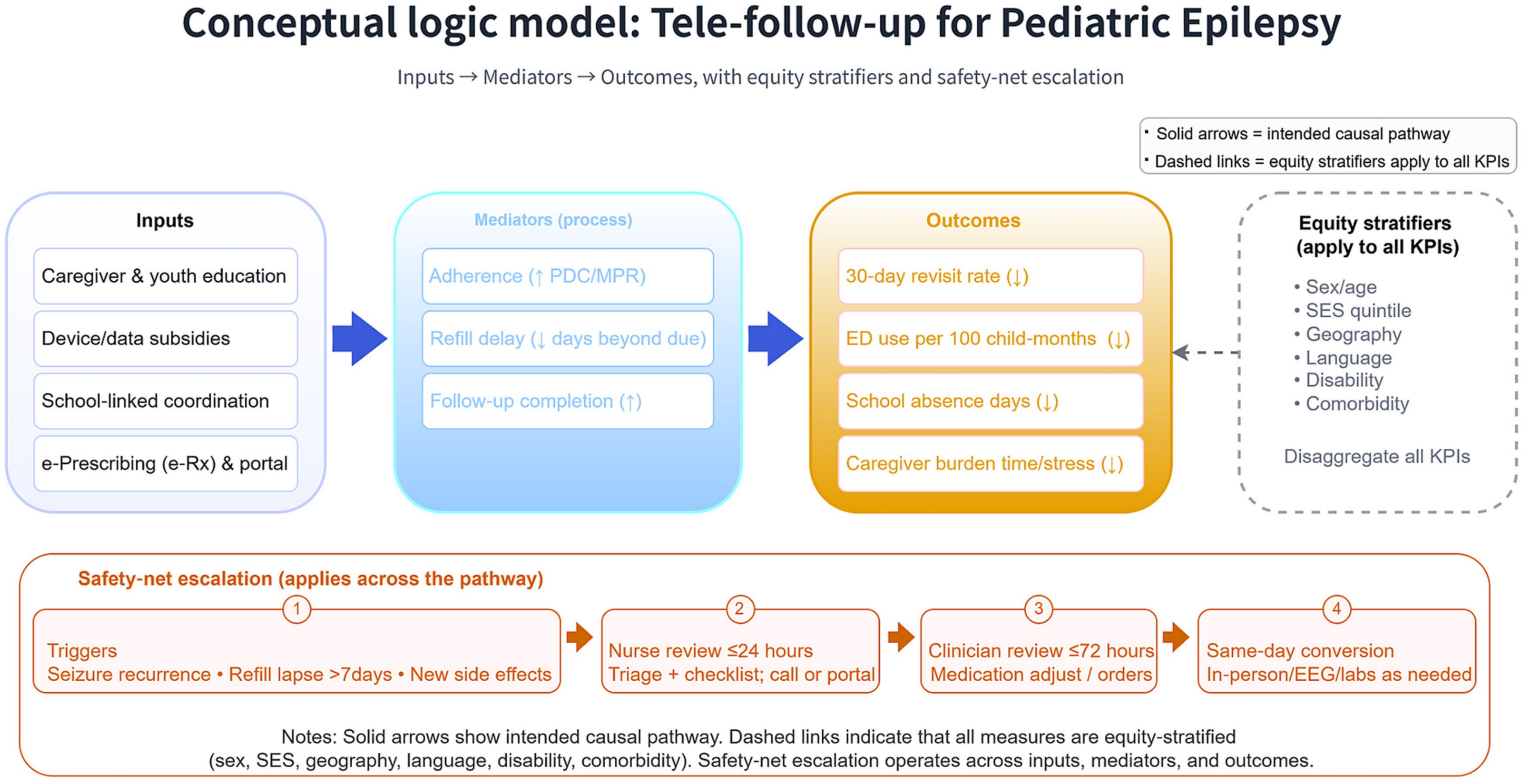
Conceptual logic model for tele-follow-up in pediatric epilepsy. Inputs (education, device/data subsidies, school-linked coordination, e-prescribing/portal) influence mediators (adherence ↑, refill delay ↓, follow-up completion ↑), leading to outcomes (30-day revisit rate ↓, ED use per 100 child-months ↓, school-absence days ↓, caregiver burden ↓). Safety-net escalation operates across the pathway; equity stratifies (sex/age, SES, geography, language, disability, comorbidity) apply to all KPIs. PDC, proportion of days covered; MPR, medication possession ratio; ED, emergency department; e-Rx, electronic prescribing; KPI, key performance indicator; SES, socioeconomic status; EEG, electroencephalography.

Figure 1 shows a logic model (inputs, mediators, outcomes)—with safety-net escalation across the pathway and equity stratifiers applied to all key performance indicators (KPIs).

### 1.1 Search strategy and selection criteria

We searched PubMed and Scopus (Jan 1, 2019–Oct 18, 2025) using terms combining “pediatric epilepsy” with “telehealth/telemedicine/remote follow-up/e-prescribing/adherence.” We prioritized pediatric studies and major guidelines/consensus (ILAE, NICE, WHO, UNICEF/ITU, OECD) relevant to follow-up, adherence/refills, unplanned care, equity, privacy, and implementation/KPIs. Adult-only diagnostic papers and studies without follow-up outcomes were excluded unless directly informative. Evidence was narratively synthesized; no meta-analysis was performed.

## 2 Evidence signals from tele-follow-up

### 2.1 Adherence and refill timeliness

Across pediatric epilepsy services, tele-follow-up strengthens continuity between in-person visits rather than replacing hands-on assessment. Randomized adherence trials are scarce, but real-world programs show higher process fidelity (visit completion, guideline documentation) and more structured medication review during remote visits; a multicenter study documented uptake of Epilepsy Quality Metrics, including safety counseling (9). Guidance endorses telehealth for dose titration, adverse-effect checks, seizure-diary review, and counseling when examination needs are limited (1, 6). Timely specialist access after index events is associated with lower downstream utilization, supporting tele-enabled rapid-follow-up pathways that close treatment and refill gaps (4). The American Epilepsy Society reports better adherence to standardized quality measures in telehealth—including audio-only—when embedded in team-based workflows (5). Small pediatric series show high completion and satisfaction with virtual maintenance visits, with stable time-to-follow-up (10, 11). Overall, direct refill-gap outcomes remain limited, but convergent evidence from quality-metric audits and access-timeliness cohorts suggests tele-follow-up reduces operational drivers of delayed refills when protocols are explicit and pharmacy links are integrated (4, 5, 9).

### 2.2 Unplanned care and emergency department (ED) utilization

Beyond adherence gains, several cohorts suggest tele-follow-up can reduce unplanned care, although findings are mixed. In a pediatric tuberous sclerosis complex cohort (1,206 visits), telemedicine exposure was associated with fewer emergency visits and hospitalizations, particularly with higher tele-visit frequency, implying dose–response effects of proactive remote management (12). Pandemic-era surveillance in the United States showed a decline in seizure-related ED visits, reflecting both behavior change and substitution by remote care; however, attribution to telehealth alone is uncertain (13). In pediatric neurology programs that rapidly virtualized, clinicians rated most encounters satisfactory with low short-interval conversion to in-person visits, suggesting stability of near-term risk (10). Other large child-neurology datasets document neutral differences in acute utilization across telehealth vs. in-person periods after reopening, indicating that context and case mix matter (11). Moderators likely include disease severity, caregiver health literacy, and digital access (video vs. audio), which can affect triage accuracy and follow-through on action plans (7, 14). Taken together, tele-follow-up appears noninferior—and sometimes favorable—for 30-day unplanned care in stable pediatric epilepsy populations, but program-level monitoring is essential to detect rebound ED revisits in higher-risk groups.

### 2.3 Hybrid and risk-stratified schedules

Hybrid models combining an in-person baseline assessment (neurologic exam, EEG, growth/development checks) with primarily remote maintenance visits have become pragmatic standards. Guidelines endorse telehealth for ongoing review in well-characterized patients, reserving in-person evaluation for new diagnoses, breakthrough seizures, suspected adverse events, regression, or comorbidity concerns (1, 6). Real-world child-neurology analyses indicate that epilepsy and ADHD were disproportionately managed by telemedicine once clinics reopened, whereas neuromuscular and movement disorders required in-person assessment—an empirical foundation for stratification (11). Early follow-up access after first seizure also associates with lower subsequent utilization, supporting rapid virtual “first follow-up” pathways that route to in-person testing when red flags surface (3, 4). Programs should define and operationalize eligibility tiers (e.g., seizure-free on stable doses; controlled focal epilepsy with reliable caregivers) and automatic triggers for in-person reassessment (new events, medication changes, developmental or behavioral concerns), aligning with specialty-center standards and implementation experience from neurology telehealth (15, 16).

### 2.4 Family education and self-management components

Tele-follow-up works best when coupled with structured education and self-management. Evidence syntheses and pediatric studies report reduced caregiver anxiety and high acceptance when remote visits standardize medication logs, seizure action plans, and home video capture of events (10, 14). Reviews of telemedicine in epilepsy detail use cases for counseling, comorbidity screening, and integration of digital seizure diaries and portals to streamline side-effect reporting and reinforce rescue plans (7). Educational frameworks tailored to children and adolescents—delivered via portals/apps and reinforced during tele-visits—improve knowledge and self-efficacy, key precursors to adherence (17). International guidance (International League Against Epilepsy [ILAE]) provides practical checklists for remote neurological assessment and family-focused counseling, including what to ask and when to escalate (6). Embedding these components into follow-up templates (pre-visit questionnaires, medication reconciliation, diary review) strengthens safety-netting between clinic contacts.

### 2.5 External validity and generalizability

Most pediatric epilepsy tele-follow-up evidence comes from real-world evaluations (rapid-cycle implementations, multicenter cohorts) and pandemic-era natural experiments, with fewer randomized or pragmatic trials. Designs such as interrupted time series and large observational cohorts (with reopening comparators) offer policy-relevant estimates but can be affected by selection bias, confounding by indication, and concurrent system changes (10, 11, 16). Heterogeneity in modality, platforms, and documentation, along with digital inequities (device/broadband, language, disability), limits generalizability and underscores the need for routine KPI dashboards. Programs should track show rates, refill lag, time-to-review after breakthrough seizure, 30-day ED revisits, adverse-event capture, and validated patient-reported outcomes—stratified by severity and social risk (1, 5, 9, 13). As services normalize post-pandemic, hybrid, risk-stratified schedules anchored in these KPIs—and periodically audited for disparities—offer the most reproducible path to safe scale-up (3, 15, 16). Yet averages can mask large inequities; connectivity, language, and care complexity shape who benefits from tele-follow-up. The next section therefore addresses privacy and equity guardrails and mechanisms for course correction.

## 3 The double-edged digital divide: privacy and equity

### 3.1 Privacy-by-design and child data governance

Tele-follow-up must embed privacy by design: developmentally appropriate consent/assent, data minimization, purpose limitation, end-to-end encryption, role-based access, and auditable logs. Global frameworks converge—WHO digital health guidance (proportionality, security, equity assessment), UNICEF–ITU Child Online Protection (safety-by-default, age-appropriate terms, redress pathways), and OECD recommendations (risk mitigation without excluding children) (18–21). ILAE checklists add epilepsy-specific practices such as secure seizure-video sharing and clear documentation of who may view recordings (6). In practice, default to HIPAA- or GDPR-aligned platforms, provide plain-language consent that explains retention, secondary use, and revocation, and conduct routine privacy audits and breach drills.

### 3.2 Coverage gaps in low-connectivity/low-income groups

Connectivity gaps remain the rate-limiting step. Multiregional studies show rural, low-income, and linguistically diverse families have lower telehealth completion or rely on audio-only, reflecting disparities in broadband, devices, and digital skills (22–24). Interrupted-time and cohort analyses indicate expansion alone does not close gaps: areas with slower internet and lower socioeconomic status (SES) sustained lower primary-care engagement despite remote options (23). Within integrated systems, rural patients used different modalities and had lower follow-through, shaped by geography and work constraints (24). For children with epilepsy—who often need assistive technologies and coordinated care—affordability and access barriers track with income and disability, especially in LMICs (25). Without mitigation, tele-follow-up risks reproducing structural inequities, as pandemic syntheses emphasize (26).

### 3.3 Heterogeneous benefits

Tele-follow-up benefits are uneven. Evidence indicates that stable, well-characterized patients (e.g., seizure-free on stable doses) achieve equivalent remote care, while new diagnoses, frequent dose changes, regression, or complex neurobehavioral comorbidities require lower thresholds for in-person reassessment (6–8). Comparative studies show telemedicine is suitable for medication review and adverse-effect checks but limited for examination and for rapid EEG/imaging access when red flags emerge (7, 8). Benefits also vary by resources and literacy: families with limited connectivity or health literacy derive less value from video encounters, supporting flexible modality choice and navigator support.

### 3.4 Equity levers

Closing the divide requires practical levers. (1) Reduce access costs via device-loan programs and zero-rated data or prepaid vouchers through public–private partnerships, aligned with WHO–UNICEF assistive-technology guidance (25). (2) Provide offline/near-point hubs—primary care, schools, community sites—with private rooms, connectivity, and staff who can assist with portals and forms; integrate school-linked supports (medication logs, rescue-plan drills) to backstop daily management. (3) Deliver multilingual, culturally adapted materials—action plans, side-effect checklists, portal instructions—co-designed with caregivers, following UNICEF–ITU and OECD principles of transparency and inclusivity (19, 21). Standardize inclusive workflows from ILAE and WHO guidance—pre-visit questionnaires, secure video instructions, accessible consent formats, disability on-ramps—to reduce friction at each step (18–21).

### 3.5 Measurement of inequity

Equity must be measured, not assumed. Disaggregate core KPIs by sex, age, disability, language, SES quintile, and urban–rural status: show rate, refill lag, time-to-review after breakthrough seizure, 30-day ED revisits, adverse-event capture, and patient-reported outcomes. Track absolute and relative differences; summary indices such as SII and RII allow comparison across time and sites (27). WHO digital health guidance recommends routine equity dashboards that flag widening gaps and prompt corrective actions (e.g., device/data subsidies, interpreter-first scheduling) (18). Transparent reporting to institutional governance and family advisory councils aligns incentives with equity performance.

Beyond near-term metrics (visit completion, revisits), evaluation should track behavior-linked outcomes: caregiver first-aid knowledge, medication adherence, navigation confidence, school attendance, and developmental trajectories. Stratifying these outcomes indicates whether tele-follow-up is closing or widening gaps in self-management and life-course opportunities.

The surest way to embed equity is through visible, comparable, and trackable metrics. The next section therefore outlines an operational KPI framework stratified by priority populations.

## 4 Monitoring quality and safety with equity-stratified KPIs

### 4.1 Evaluation methods that matter

Implementation-science frameworks help tele-follow-up mature from pilots to accountable services. The RE-AIM model highlights reach (equitable access), effectiveness (adherence, time-to-review), adoption across clinics, workflow fidelity (triage, escalation), and long-term maintenance—domains easily mapped to pediatric epilepsy dashboards (28). The Consolidated Framework for Implementation Research adds determinants across intervention design (templates, escalation rules), inner setting (staffing, pharmacy links), outer setting (payer rules), individual factors (caregiver digital literacy), and implementation processes (audit-and-feedback) (29). For day-to-day oversight, use run charts; statistical process control adds rules to separate common from special-cause variation (30). When policies or workflows change (e.g., hybrid scheduling, refill reminders), apply interrupted time series for level/slope shifts and difference-in-differences against similar services that did not change (31, 32). WHO and AHRQ guidance favors pragmatic, frequent, longitudinal measurement embedded in routine care—rather than sporadic audits—especially while datasets evolve (33, 34).

### 4.2 KPI framework and data plumbing

Effective monitoring depends on robust data flows. Core sources include the EHR (appointments, demographics, comorbidities), e-prescribing/pharmacy fills, ED/hospital encounters, and portals (diaries, PROs). Pre-specify reproducible measures and equity stratifiers, and maintain all definitions in Table 1. Build a unified dashboard that ingests all streams and uses consistent visit-modality labels. Common failure points—claims lags, siloed ED data, inconsistent coding—erode comparability; align definitions with NICE and ILAE standards (1, 6). OECD health-data governance principles (secure linkage, proportionate use, clarity of purposes) are prerequisites for trustworthy KPI reporting at scale (20).

**Table 1:** KPI and inequity panel (operational specification).

Indicator	Definition (num/den/window)	Data source	Stratifiers (apply to all)	Suggested target	Use/action
Tele-follow-up completion rate	Completed tele-visits ÷ scheduled tele-visits; monthly, roll-up quarterly	Scheduling system; platform logs; EHR status	Sex/age; SES quintile; geography; language; connectivity; disability; comorbidity	Stable ≥85%; High-risk ≥90%	Run chart; funnel by clinic; capacity tuning
Time-to-review after breakthrough seizure	Median days from index event → clinician review; monthly	PRO/helpline; ED dx codes; EHR alerts	Same as above	Stable ≤7 d; High-risk ≤3 d	SPC; escalation SLA monitoring
30-day revisit rate (post-tele-visit)	Patients with ≥1 epilepsy-related revisit within 30 d ÷ tele-visit completions; monthly	EHR encounters; dx codes	Same as above	≤8% overall; quarterly ↓ in high-risk	Signal of safety; triggers case audit
ED visits per 100 child-months	ED epilepsy visits ÷ child-months ×100; monthly/quarterly	EHR + (HIE if available)	Same as above	Downward trend QoQ	Burden normalization; seasonality check
Adherence (PDC ≥ 80%)	Patients with PDC ≥ 80% ÷ eligible; quarterly	e-Rx; pharmacy/claims; EHR e-prescribing	Same as above	≥75% overall; ≥70% high-risk and improving	Medication management; outreach targeting
e-Rx turnaround time	Median hours from e-Rx signed → pharmacy ready	EHR timestamps; pharmacy ACK	Same as above	P50 ≤ 24 h; P90 ≤ 72 h	Bottleneck triage; payer/PA negotiation
No-show rate (tele)	No-shows ÷ scheduled tele-visits; monthly	Scheduling + platform check-in	Same as above + timeslot (school day/weekend/evening)	≤12%; weak-connectivity gap ≤1.3×	Reminder design; slot redesign
Equity gap in completion	Abs diff + RII (top vs. bottom SES quintile); quarterly	KPI + geo/SES linkage	—	Abs diff ≤5 pp; RII ≤ 1.15	Executive equity dashboard; accountability

1. Index event (Time-to-review): earliest of caregiver/patient report → ED epilepsy dx → EHR auto-flag (EEG/med change); use earliest timestamp. 2. Child-months example: 200 children × 6 months = 1,200 child-months; 36 ED visits ⇒ 36/1,200 × 100 = 3.0 per 100 child-months. 3. RII = rate (top SES quintile) ÷ rate (bottom SES quintile); pp = percentage points. ACK, acknowledgment; d, days; dx, diagnosis; ED, emergency department; e-Rx, electronic prescribing; EHR, electronic health record; HIE, health information exchange; KPI, key performance indicator; P50, 50th percentile; P90, 90th percentile; PA, prior authorization; PDC, proportion of days covered; pp., percentage points; QoQ, quarter-over-quarter; RII, relative index of inequality; SII, slope index of inequality; SLA, service-level agreement; h, hours; PRO, patient-reported outcome.

Table 1 outlines the KPI panel—definitions, data sources, stratifiers, targets, and use.

Implementing such monitoring systems in primary-care or district settings will inevitably face constraints in workforce, connectivity, and data systems. Teams may therefore start with a small set of high-yield KPIs, focus on a few key equity stratifiers, and review results quarterly rather than monthly, adapting targets to what is feasible in their context.

### 4.3 Safety nets and escalation thresholds

Quality monitoring requires explicit safety nets. Automate prompts for seizure recurrence/clusters; refill lapse >7 days; new adverse effects (e.g., rash, behavior change); any unplanned ED/hospital visit; or caregiver-reported declines in sleep, mood, or school function.

Set service-level response targets: nurse review ≤24 h; clinician review ≤72 h; same-day conversion (video or in-person) for breakthrough seizures in newly diagnosed or drug-resistant epilepsy. Define rapid in-person fallbacks (EEG/labs) and direct-to-ED criteria.

Use nurse-led triage with structured scripts; pre-visit questionnaires can auto-score risk and trigger escalation. These practices align with ILAE and NICE risk-stratified follow-up standards; pediatric chronic-care models inform alert thresholds and callback windows (1, 6). Apply statistical process control to balance sensitivity against false alarms and iterate thresholds over time (30).

### 4.4 Exemplary KPI set (definitions and targets)

See Table 1 for operational definitions; below explains how to interpret and act on KPIs without repeating numerator/denominator/window.

- *Completion rate + Dropout* — A paired access signal. Rising dropout (missed without rescheduling) usually reflects scheduling friction, language barriers, or unstable connectivity → target outreach and slot redesign.
- *Refill delay vs Adherence* (proportion of days covered/medication possession ratio ≥80%) — Process vs. persistence. Fix system delays (e-prescribing, pharmacy, prior authorization) before expecting adherence gains; monitor the 50th and 90th percentiles to surface tail risk.
- *Time-to-review (after breakthrough seizure)* — Primary guardrail for clinical risk; confirms that escalation is acted on (targets: stable ≤7 days; high risk ≤3 days).
- *30-day revisit + ED per 100 child-months* — Safety balance check. Stable revisits with falling ED/child-months ⇒ safer community management; the reverse pattern ⇒ case audit and threshold tuning.
- *Equity view* — Stratify every KPI (sex/age, SES, geography, language, disability, comorbidity). Track absolute gaps and the relative and slope indices of inequality (relative index of inequality/slope index of inequality) with named owners and closure timelines.
- *Run-time analytics* — Use run charts/Statistical process control for routine oversight; reserve interrupted time series/difference-in-differences for major policy/workflow shifts to separate signal from noise and attribute effects (30–32).
- *Illustrative vignette* — For example, a regional clinic may notice that overall tele-visit completion is high but markedly lower for rural families without broadband. An equity-stratified run chart prompts root-cause discussions that identify connectivity problems and inconvenient appointment times. The team then tests simple changes—SMS reminders, nurse-initiated calls, and evening slots—and uses the same KPIs to confirm that missed visits in this subgroup fall over the next 6 months.
- *Auto-triggers* — Over-target time-to-review, rising revisits, upward ED trends, refill lapse >7 days, or widening equity gaps should auto-flag: nurse review → clinician review → same-day conversion (video/in person) when indicated (see Section 4.3).

### 4.5 Budget impact and payer alignment

Budget impact analysis estimates near-term affordability: define the eligible population, expected tele-follow-up uptake, unit costs (tele-visits, nurse triage, dashboard maintenance), and offsets (reduced travel, missed school/work, preventable ED/hospitalizations). Sensitivity analyses should vary uptake, ED rates, and device/data subsidies.

Sustainability rests on incentives: value-based or hybrid payment models can link reimbursement to key tele-follow-up KPIs (e.g., time-to-review, ED revisits, adherence) (35). U. S. Medicare’s FY 2025 IPPS/LTCH rule provides precedents for tying payment and quality programs to performance measures; OECD frameworks support cross-system benchmarking and governance for digital indicators (20, 35). Reimbursement contracts should explicitly require equity-stratified reporting and remediation plans when performance gaps widen.

*Integrative note.* Equity-stratified KPI oversight—implemented with pragmatic methods, reliable data plumbing, explicit safety nets, and aligned incentives—turns tele-follow-up from a collection of virtual visits into a measurable, trustworthy service for children with epilepsy.

## 5 Discussion and future directions

Across pediatric epilepsy care, tele-follow-up is best used as a complement—not a wholesale substitute—for in-person services. When embedded in structured pathways, medication review, seizure-diary reconciliation, and counseling are often noninferior to clinic visits, with signals for faster post-index follow-up and fewer refill gaps; effects, however, vary by site, population, and baseline access (4, 8). Contemporary guidance endorses risk-stratified, continuity-focused schedules (e.g., stable patients alternating remote and in-person; expedited face-to-face for new diagnosis, breakthrough seizures, or notable adverse effects) (1). Since 2020, policy has normalized telehealth, yet heterogeneity in outcomes likely reflects differences in digital access, health literacy, and local workflows rather than the modality itself (26). The practical question therefore is not whether tele-follow-up “works,” but for whom, under what conditions, and with what guardrails.

Programs should treat privacy-by-design and equity as core quality domains. Foundational controls—child-appropriate consent, data minimization, encryption, and role-based access—align with the WHO digital health strategy and UNICEF/ITU child-online protection guidance (19, 34). Operationally, the digital divide remains rate-limiting: connectivity constraints, device scarcity, language barriers, and caregiver work schedules predict tele-dropout and missed escalations. Mitigations include device/data subsidies, multilingual materials, and offline handover points via schools or primary care; these require clear governance and financing. Research priorities include: (i) long-term pediatric outcomes in low-connectivity or low-income settings; (ii) privacy-impact evaluations for seizure videos and patient portals (including cross-border storage); and (iii) interoperability that links e-prescribing/pharmacy claims, ED data, and patient-reported outcomes to detect refill lapses and breakthrough seizures in near real time. Pragmatic trials and mixed-methods platform evaluations should embed equity endpoints (SES quintile, geography, language, disability, digital literacy) and report effect heterogeneity, not averages alone (4, 8, 19, 34).

Looking ahead, health systems should adopt standardized, risk-stratified schedules anchored in NICE/ILAE content for remote visits; deploy equity-stratified KPI dashboards (show rate, refill lag, time-to-review, 30-day revisits, ED visits per 100 child-months) for transparency and cross-site benchmarking; and strengthen school-linked touchpoints to support daily management (1). Durability will hinge on payer alignment: value-based or bundled/hybrid payments can tie reimbursement to quality and equity performance (e.g., improvements in adherence or time-to-review among the lowest-SES quintiles), consistent with recent policy and international analyses (33, 36). Standard-setting bodies should specify minimum disaggregation for public reporting and require remediation when gaps widen. Ultimately, tele-follow-up must evolve from *ad hoc* innovation to a measured, equitable, and accountable pediatric epilepsy infrastructure—balancing efficiency with inclusiveness, and privacy with actionable data—so families gain continuity without compromising safety.
